# Synthesis, Crystal Structures, and Optical and Magnetic Properties of Samarium, Terbium, and Erbium Coordination Entities Containing Mono-Substituted Imine Silsesquioxane Ligands

**DOI:** 10.1021/acs.inorgchem.2c04371

**Published:** 2023-01-30

**Authors:** Patrycja Wytrych, Józef Utko, Mariusz Stefanski, Julia Kłak, Tadeusz Lis, Łukasz John

**Affiliations:** † Faculty of Chemistry, University of Wrocław, 14 F. Joliot-Curie, 50-383Wrocław, Poland; ‡ Institute of Low Temperature and Structure Research, Polish Academy of Sciences, 2 Okólna, 50-422Wrocław, Poland

## Abstract

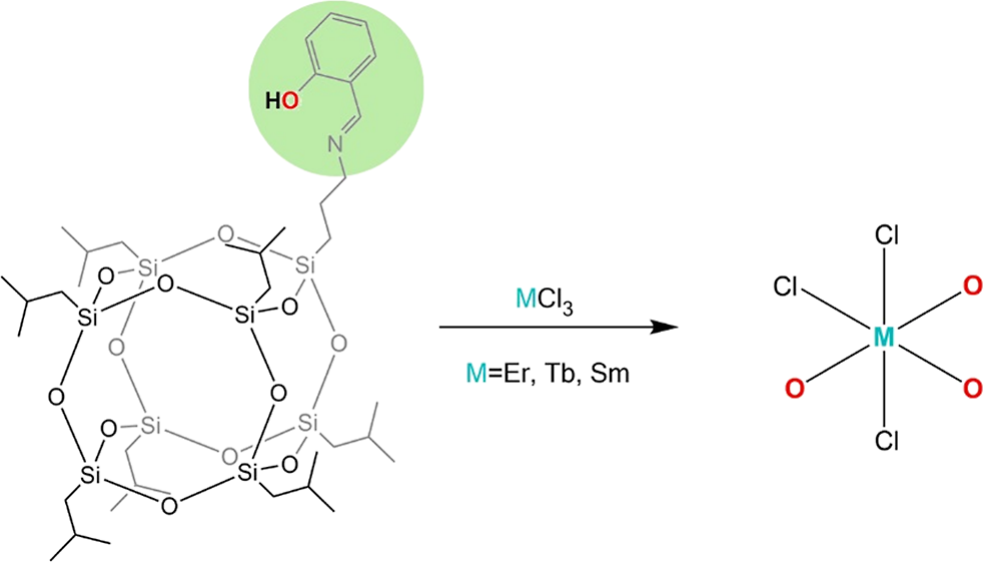

Mono-substituted cage-like silsesquioxanes of the T_8_-type can play the role of potential ligands in the coordination chemistry. In this paper, we report on imine derivatives as ligands for samarium, terbium, and erbium cations and discuss their efficient synthesis, crystal structures, and magnetic and optical properties. X-ray analysis of the lanthanide coordination entities [MCl_3_(POSS)_3_]·2THF [M = Er^3+^ (**3**), Tb^3+^ (**4**), Sm^3+^ (**5**)] showed that all three compounds crystallize in the same space group with similar lattice parameters. All compounds contain an octahedrally coordinated metal atom, and additionally, **3** and **5** structures are strictly isomorphous. However, surprisingly, there are two different molecules in the crystal structure of the terbium coordination entity **4**, monomer (sof 65%) and dimer (sof 35%), with one and two metal centers. Absorption measurements of the investigated materials recorded at 300 K showed that regardless of the lanthanide involved, their energy band gap equals 2.7 eV. Moreover, the analogues containing Tb^3+^ and Sm^3+^ exhibit luminescence typical of these rare earth ions in the visible and infrared spectral range, while the compound with Er^3+^ does not generate any emission. Direct current variable-temperature magnetic susceptibility measurements on polycrystalline samples of **3–5** were performed between 1.8 and 300 K. The magnetic properties of **3** and **4** are dominated by the crystal field effect on the Er^3+^ and Tb^3+^ ions, respectively, hiding the magnetic influence between the magnetic cations of adjacent molecules. Complex **5** exhibits a nature typical for the paramagnetism of the samarium(III) cation.

## Abstract 2

In this paper, we report on imine-polyhedral oligomeric silsesquioxane derivatives as ligands for samarium, terbium, and erbium cations and discuss their efficient synthesis, crystal structures, and optical and magnetic properties.

## Introduction

The variety of functionalization and further modification of the organic arms anchored to the silicon atoms in the polyhedral oligomeric silsesquioxane (POSS) cages makes these compounds unique systems with a wide range of applications. One of the most exciting futures is using mono-substituted or fully substituted cage-like architectures as a new class of potential ligands in coordination chemistry. For instance, in the case of POSS-containing complexes, the most investigated are metalated T_7_ moieties of formula R_7_Si_7_O_12_M (where R = a cyclopentyl or isobutyl group; M = a d-block cation) with additional ligands coordinated to the metal-containing corner.^1−5^ Zinc(II) alkyl silsesquioxanes were reported to co-polymerize carbon dioxide and cyclohexene oxide.^6^ Tin(II), aluminum(III), gallium(III), and zirconium(IV) POSS-based species were also investigated and used, among others, as models for alkene polymerization.^7−9^ Also, silsesquioxane-like complexes containing iron,^10^ titanium,^11^ and zirconium^12^ cation connections were reported as molecular models for heterogeneous catalysis. Other exciting applications of POSS-containing coordination entities and their hybrids were found, among other things, in the area of flexible displays, light-harvesting systems, up-converters for photovoltaic materials, chemical sensors, and so forth.^13−16^

More recently, POSS-based octahedral systems were also intensively explored. Cubic metal-containing silsesquioxanes can be divided into two groups. The first includes species with a metal ion directly anchored to the inorganic core, mainly via silicon or oxygen atoms. The second group comprises seven non-reactive organic arms and one metalated corner. Such compounds possess various features. As mentioned above, the metal sites attached to cubic inorganics can constitute efficient catalytic centers and play the role of soluble models for, for example, tethered Os^4+^ and Rh^2+^ species.^17,18^ An organopalladium(II) cage-like silsesquioxane was also studied as a catalyst for the Suzuki–Miyaura coupling.^19^ Also, our group described the molecular structures of the first Pd(II) coordination entities containing mono-functionalized amine-POSS ligands examined in the C–C coupling.^20^

Furthermore, a ruthenium-containing POSS was examined in silylative couplings.^21^ There are also known Al^3+^ and Zn^2+^ POSS species active in the ROP of *rac*-lactide.^22,23^ Also, it was shown that a reaction between ZnR_2_ (R = OAc, Et) and fully substituted imine-POSS leads to a tetrazinc(II) compound. The resulting coordination entity was studied as a catalyst for converting CO_2_ and epoxides into cyclic carbonates.^24^ In turn, the Hoveyda–Grubbs olefin metathesis can be performed using a ruthenium cage-like silsesquioxane.^25^ Other metal-based silsesquioxanes active in catalytic transformations include zirconium(IV) species active in SiO_2_-supported olefin polymerization.^26^ The literature reports are relatively sparse in the case of copper-containing silsesquioxanes. For example, Shul’pin et al. reported on isomeric copper(II)-sodium silsesquioxanes as efficient catalysts in alkane oxidation with peroxides.^27^ The same group also described the synthesis of a series of copper(II) silsesquioxanes with 2,9-dimethyl-1,10-phenanthroline^28^ and hexanuclear and “peanut cage” Cu_9_-cluster-containing phenylsilsesquioxane moieties.^29,30^ Such systems can be used in the oxidation of alcohols and alkanes with peroxides, and alcohols to ketones with *tert*-butyl hydroperoxide and in hyperperoxidation of alkanes with H_2_O_2_. Crucial findings concerning the reactivity with azide ions and voltammetric application of copper-containing octakis(3-aminopropyl)octasilsesquioxane were also conveyed.^31^ Another example is mono-substituted T_8_-type cage-like silsesquioxanes bound by trifunctional acyl chloride as N,O-donor ligand for Cu^2+^ cations.^32^

Lanthanide coordination entities are attractive because of their interesting physicochemical features and numerous applications as so-called new materials.^33−35^ Incredibly, the luminescence^36^ and magnetism^37^ of 4f element coordination entities have stimulated groundbreaking discoveries in these particular areas of interest. In the case of magnetism, various lanthanide cations are perfect candidates for magnetic materials manifesting high spins and introducing anisotropy derived from the nature of the f-electron shell.^38,39^ Several complexes possessing f-block cations have been available. However, it should be emphasized that little is known about the nature of the exchange interactions between rare earth cations and other magnetic centers.^40^ Furthermore, the detailed description of the magnitude and nature of the mentioned-above interactions is complex due to the significant orbital contributions dealing with lanthanide ions and the specific effects of the crystal field.^41^

We herein report on less-explored imine derivatives of mono-substituted POSSs applied as ligands for samarium, terbium, and erbium cations and discuss their efficient synthesis, crystal structures, and magnetic and optical properties.

## Experimental Section

### Materials

Trisilanolisobutyl-POSS (Hybrid Plastic, USA), tetramethylammonium hydroxide (25% in methanol, Alfa Aesar), and (3-aminopropyl)triethoxysilane (98%, Alfa Aesar) were purchased. Salicylaldehyde (98%), anhydrous erbium chloride (99.9%), anhydrous terbium chloride (99.9%), and anhydrous samarium chloride (99.9%) were purchased from Sigma-Aldrich (Darmstadt, Germany) and used without further purification. Ethanol (HPLC grade, J. T. Baker, Gdańsk, Poland), methanol (HPLC grade, J. T. Baker, Gdańsk, Poland), acetonitrile (HPLC grade, J. T. Baker, Gdańsk, Poland), toluene (HPLC grade, J. T. Baker, Gdańsk, Poland), and tetrahydrofuran (Chempur) were purchased. All solvents, except methanol and acetonitrile, were used with further purification. Tetrahydrofuran and toluene were purified by distillation with sodium wires, and ethanol was purified with metallic magnesium.

### Methods

^1^H, ^13^C, and ^29^Si NMR spectra were recorded using a Bruker Avance 500 or equipped with broadband inverse gradient probe heads. All spectra were collected at 500 MHz using a relaxation delay of 1.0 s and a pulse width of 7°. Spectra were referred to the residual solvent signal (CDCl_3_ for ^1^H NMR and ^13^C NMR) or TMS (0.00 ppm for ^29^Si NMR) as an internal reference. FT-IR spectra of all compounds were measured using a Bruker Vertex 70 FTIR spectrometer. Samples spectra were recorded as KBr pellets. The FT-IR sample chamber was flushed continuously with N_2_ prior to data acquisition in the range 4000–400 cm^–1^ with a precision of ±1 cm^–1^. Elemental analyses (C, H, N, Cl) were performed using a Vario EL III element analyzer (Hanau, Germany). Quantitative analyses of Si, Er, Tb, and Sm were performed using an emission spectrometer iCAP 7400 DUO icp–Thermo Fisher Scientific (Waltham, MA, USA). Diffraction data for all resulting coordination entities were collected at 100 K on monocrystalline diffractometer with a XtaLAB Synergy R, DW system HyPix-Arc 150 κ-axis four-circle diffractometer with mirror-monochromated Mo Kα radiation. The CrysAlisPro software from Oxford Diffraction was used to determine cell parameters, data reduction, and absorption correction for all crystals. The structures were solved by direct methods (SHELXS97) and refined using the least-squares technique (SHELXL2013), CCDC nos.: 2226038 (for **3**), 2226039 (for **4**), and 2226040 (for **5**). The absorption measurement was carried out in the backscattering mode using an Agilent Cary 5000 spectrophotometer. The emission spectra were collected using an FLS980 Fluorescence Spectrometer from Edinburgh Instruments. The laser diodes operating under 375 and 405 nm were used as excitation sources. The luminescence decay profiles were recorded using a femtosecond laser (Coherent Model Libra). Magnetic susceptibility and magnetization measurements were performed on a Quantum Design SQUID magnetometer (MPMS-3). Direct current (dc) magnetic measurements were carried out on polycrystalline samples of **3–5** in the 1.8–300 K temperature range with an applied external magnetic field of 0.5 T. Corrections are based on subtracting the sample-holder signal and contribution χ_D_ estimated from Pascal’s constants.^42^ The samples were restrained by adding a small amount of paraffin oil to prevent torquing.

### Syntheses

All reactions were carried out under a dinitrogen atmosphere.

#### Synthesis of **1**

**1** was prepared following a modified procedure described by X. Zhou et al.^43^ Trisilanolisobutyl-POSS (5 g, 6.32 mmol) was dissolved in an ethanol/methanol mixture (30/10 mL). When stirring, to the solution was added tetramethylammonium hydroxide (14 μL, 0.13 mmol, 0.021 equiv), followed by the dropwise addition of (3-aminopropyl)triethoxysilane (1.47 mL, 6.32 mmol, 1 equiv). The mixture was stirred at RT for 7 days. After this time, the reaction mixture was concentrated on a rotary evaporator to give a white suspension, which was precipitated with acetonitrile. Then, the resulting white solid was filtered and dried under reduced pressure to a white powder (5.36 g, 97%). ^1^H NMR (500 MHz, CDCl_3_, 300 K, ppm): δ 2.64 (t, 2H, ^3^*J*_HH_ = 7,14 Hz, CH_2_–N), 1.83 (quint, 7H, ^3^*J*_HH_ = 6,5 Hz, CH, ^*i*^Bu), 1.50 (m, 2H, CH_2_), 0.93 (dd, 42H, ^3^*J*_HH_ = 6,71 Hz, CH_3_, ^*i*^Bu), 0.58 (m, 16H, two signals 14H and 2H overlapped, SiCH_2_+ SiCH_2_, ^*i*^Bu). ^13^C NMR (500 MHz, CDCl_3_, 300 K): δ 44.92 (CH_2_–N), 27.31 (CH_2_), 25.84 (CH_3_, ^*i*^Bu), 24.00 (CH, ^*i*^Bu), 22.66 (CH_2_, ^*i*^Bu), 9.41 (SiCH_2_). ^29^Si NMR (500 MHz, CDCl_3_, 300 K): δ −67.28 (SiCH_2_CH_2_CH_2_NH_2_), −67.70 (3 Si closed to amine arm), −67.89 (remaining 4 Si). FT-IR (cm^–1^, KBr): ν_NH_ 3436 (s), ν_CH_ 2955 (m), ν_NH_ 1623 (m), ν_CH_ 1466 (s), δ_CH3_ 1367 (s), ν_C–N_ 1333 (s), ν_Si–CH_ 1231 (s), ν_Si–O–Si_ 1114 (s), δ_CH_ 838 (s), δ_CH_ 746 (m), δ_O–Si–C_ 483 (s). Elemental analysis (%) for C_31_H_71_NO_12_Si_8_: calcd C 42.57, H 8.18, N 1.60, Si 25.69; found, C 42.48, H 8.14, N 1.53, Si 25.64.

#### Synthesis of **2**

Imine-functionalized POSS **2** was prepared following a modified procedure described by Jones and Mahon et al.^23^**1** (2.00 g, 2.29 mmol) was dissolved in toluene (30 mL), and salicylaldehyde (0.30 mL, 2.86 mmol, 1.25 equiv) was added. The reaction was performed at RT for 24 h. The resulting yellow suspension was then filtered and concentrated under vacuum. A yellow oil was obtained, which was recrystallized from EtOH, leading to precipitation. The precipitate was filtered and dried in vacuo to give a yellow powder of **2** (1.96 g, 87.5% yield). ^1^H NMR (500 MHz, CDCl_3_, 300 K): δ 8.32 (s, 1H, N=CH), 7.30 (td, 1H, ^3^*J*_HH_ = 8.68 Hz, Ar), 7.23 (dd, 1H, ^3^*J*_HH_ = 7.71 Hz, Ar), 6.97 (d, 1H, ^3^*J*_HH_ = 8.16 Hz, Ar), 6.87 (td, 1H, ^3^*J*_HH_ = 7.35 Hz, Ar), 3.58 (t, 2H, ^3^*J*_HH_ = 6.77 Hz, CH_2_), 1.85 (m, 9H, CH, ^*i*^Bu), 0.95 (m, 42H, CH_3_, ^*i*^Bu), 0.67 (m, 2H, Si–CH_2_), 0.61 (m, 14H, Si–CH_2_). ^13^C NMR (500 MHz, CDCl_3_, 300 K): δ 164.84 (CH, C=N), 161.65 (C, Ar–OH), 132.19 (Ar), 131.32 (Ar), 118.99 (Ar), 118.52 (Ar), 117.20 (Ar), 62.21 (CH_2_–N), 25.84 (CH_3_, ^*i*^Bu), 24.54 (CH_2_), 24.04 (CH, ^*i*^Bu), 22.66 (CH_2_, ^*i*^Bu), 9.80 (Si–CH_2_). ^29^Si NMR (500 MHz, CDCl_3_, 300 K): δ −67.28 (SiCH_2_CH_2_CH_2_NH=C), −67.70 (3 Si close to functionalized arm), −67.89 (remaining 4 Si). FT-IR (cm^–1^, KBr): ν_OH_ 3435 (s), ν_CH_ 2955 (m), ν_C=N_ 1635 (s), δ_N–H_ 1585 (m), ν_CH_ 1466 (s), δ_CH3_ 1367 (s), ν_C–N_ 1334 (s), ν_C–O_ 1282 (s) ν_Si–CH_ 1231 (s), ν_Si–O–Si_ 1112 (s), δ_CH_ 838 (s), δ_CH_ 746 (m), δ_O–Si–C_ 483 (s). Elemental analysis (%) for C_38_H_75_NO_13_Si_8_: calcd C 46.63, H 7.72, N 1.43, Si 22.96; found, C 46.58, H 7.62, N 1.37, Si 22.91.

#### Synthesis of **3**

Anhydrous erbium chloride (0.12 g, 0.45 mmol) and **2** (1.31 g, 1.34 mmol, 3 equiv) were dissolved in THF (30 mL). The reaction mixture was stirred at RT for 24 h. The resulting yellow suspension was then filtered and concentrated to ca. 10 mL. The solution was stored in a refrigerator. After about 10 days, colorless crystals of **3** were obtained (1.31 g, 91%). FT-IR (cm^–1^, KBr): ν_OH_ 3435 (s), ν_CH_ 2955 (m), ν_C=N_ 1659 (s), δ_N–H_ 1610 (s), δ_C=C_ 1540 (s), ν_CH_ 1467 (s), δ_CH3_ 1367 (s), ν_C–N_ 1334 (s), ν_C–O_ 1292 (s) ν_Si–CH_ 1231 (s), ν_Si–O–Si_ 1109 (s), δ_CH_ 838 (s), δ_CH_ 741 (s), δ_O–Si–C_ 481 (s). Elemental analyses (%) for ErCl_3_(C_38_H_75_NO_13_Si_8_)_3_(C_4_H_8_O): calcd C 43.19, H 7.16, N 1.28, Cl 3.24, Er 5.10, Si 20.54; found, C 43.01, H 7.02, N 1.19, Cl 3.21, Er 5.02, Si 20.49.

#### Synthesis of **4**

Anhydrous terbium chloride (0.083 g, 0.31 mmol) and **2** (0.91 g, 0.93 mmol, 3 equiv) were dissolved in THF (30 mL). The reaction mixture was stirred at RT for 24 h. The resulting yellow suspension was then filtered and concentrated to ca. 10 mL. The solution was stored in the refrigerator. After about 10 days, colorless crystals of **4** were obtained (0.90 g, 88%). FT-IR (cm^–1^, KBr): ν_OH_ 3435 (s), ν_CH_ 2955 (m), ν_C=N_ 1657 (s), δ_N–H_ 1610 (s), δ_C=C_ 1540 (s), ν_CH_ 1467 (s), δ_CH3_ 1367 (s), ν_C–N_ 1333 (s), ν_C–O_ 1291 (s) ν_Si–CH_ 1231 (s), ν_Si–O–Si_ 1107 (s), δ_CH_ 838 (s), δ_CH_ 741 (s), δ_O–Si–C_ 482 (s). Elemental analyses (%) for TbCl_3_(C_38_H_75_NO_13_Si_8_)_3_(C_4_H_8_O): calcd C 43.30, H 7.17, N 1.28, Cl 3.25, Tb 4.86, Si 20.59; found, C 43.49, H 7.01, N 1.21, Cl 3.18, Tb 4.76, Si 20.53.

#### Synthesis of **5**

Anhydrous samarium chloride (0.073 g, 0.28 mmol) and **2** (0.83 g, 0.85 mmol, 3 equiv) were dissolved in THF (30 mL). The reaction mixture was stirred at RT for 24 h. The resulting yellow suspension was then filtered and concentrated to ca. 10 mL. The solution was stored in the refrigerator. After about 10 days, colorless crystals of **5** were obtained (0.85 g, 92%). FT-IR (cm^–1^, KBr): ν_OH_ 3430 (s), ν_CH_ 2955 (m), ν_C=N_ 1656 (s), δ_N–H_ 1610 (s), δ_C=C_ 1540 (s), ν_CH_ 1466 (s), δ_CH3_ 1367 (s), ν_C–N_ 1333 (s), ν_C–O_ 1290 (s) ν_Si–CH_ 1231 (s), ν_Si–O–Si_ 1108 (s), δ_CH_ 838 (s), δ_CH_ 741 (s), δ_O–Si–C_ 482 (s). Elemental analyses (%) for SmCl_3_(C_38_H_75_NO_13_Si_8_)_3_(C_4_H_8_O): calcd C 43.41, H 7.19, N 1.29, Cl 3.26, Sm 4.61, Si 20.64; found, C 43.32, H 7.08, N 1.22, Cl 3.21, Sm 4.55, Si 20.61.

## Results and Discussion

### Synthesis and Crystal Structures

**Scheme 1:**
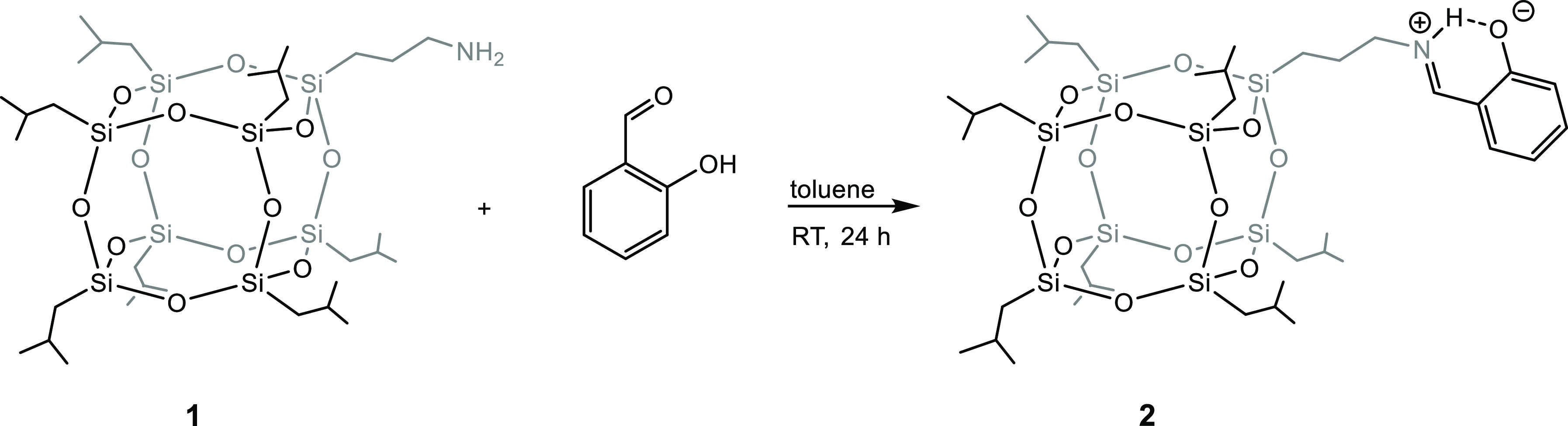
Synthesis of Imine-POSS Ligand **2**

In this study, we focused on imine-POSS-based coordination entities containing Er^3+^, Tb^3+^, and Sm^3+^ cations with well-defined crystal structures. In the first step, an imine silsesquioxane ligand **2** was obtained in the reaction between 3-aminopropylheptaisobutyl-POSS **1** with salicylaldehyde (Scheme 1).

In the next step, we reacted **2** and anhydrous lanthanide chlorides LnCl_3_ (Ln = Er^3+^, Tb^3+^, Sm^3+^) in a 3:1 molar ratio. Reactions were performed in tetrahydrofuran at room temperature for 24 h. All reactions led to the isolation of three lanthanide complexes of the formula [MCl_3_(POSS)_3_]·2THF (M = Er (**3**), Tb (**4**), and Sm (**5**)) in a crystalline form with 91, 88, and 92% yields for **3**, **4** and **5**, respectively.

The structure of **2** was unambiguously confirmed by multinuclear NMR analyses (^1^H, ^13^C, and ^29^Si), infrared spectroscopy, and elemental analysis. The chemical shifts of ^29^Si NMR signals within the expected region for mono-alkyl-substituted T_8_ cage-like silsesquioxanes at δ = −67.28, −67.70, and −67.89 ppm for one Si^*n*^PrNH=C functionalized arm, three Si atoms which are close to reactive organic arm, and four remaining silicon nuclei, respectively, confirmed the presence of three kinds of Si atoms forming an inorganic silsesquioxane core. Moreover, the characteristic peak at δ = 8.32 ppm in the ^1^H NMR spectra indicated the presence of an N=CH fragment belonging to one imine arm attached to a cage-like core. Also, the presence of the N=C bond was confirmed by ^13^C NMR (chemical shift at 164.84 ppm). Moreover, the ν(O–H) vibration from the salicylimine fragment was observed at 3435 cm^–1^.

The structures of **3–5** coordination entities were confirmed by FT-IR, elemental analysis, and X-ray studies. In the FT-IR spectra of lanthanide complexes, the Si–O–Si stretching gave strong absorptions around 1109, 1107, and 1108 cm^–1^ for **3**, **4**, and **5**, respectively. Moreover, the absorption of ν(C=N) and ν(O–H) groups was observed at 1659 and 3435, 1657 and 3435 cm^–1^, and 1656 and 3430 cm^–1^ for **3**, **4**, and **5**, respectively.

In turn, the X-ray analysis of single crystals showed that compounds [ErCl_3_(POSS)_3_]·2(C_4_H_8_O) (**3**), [TbCl_3_(POSS)_3_]·2(C_4_H_8_O) (**4**), and [SmCl_3_(POSS)_3_]·2(C_4_H_8_O) (**5**) crystallize in a triclinic system in the *P*1̅ space group. Crystallographic data for **3**, **4**, and **5** are presented in Table 1.

**Table 1:** Crystallographic Data for **3**, **4**, and **5**^a^

	**3**	**4**	**5**
Crystal Data			
chemical formula	C_114_H_225_Cl_3_N_3_O_39_Si_24_Er·2(C_4_H_8_O)	C_114_H_225_Cl_3_N_3_O_39_Si_24_Tb·2(C_4_H_8_O)	C_114_H_225_Cl_3_N_3_O_39_Si_24_Sm·2(C_4_H_8_O)
*M*_r_	3353.93	3345.59	3337.02
crystal system, space group	triclinic, *P*1̅	triclinic, *P*1̅	triclinic, *P*1̅
temperature (K)	100	100	100
*a*, *b*, *c* (Å)	16.230 (2), 22.743 (3), 27.092 (3)	16.258 (2), 22.790 (3), 27.159 (3)	16.241 (2), 22.783 (3), 27.202 (3)
α, β, γ (°)	110.97 (2), 91.51 (2), 108.79 (2)	111.10 (2), 91.42 (2), 108.75 (2)	111.19 (2), 91.62 (2), 108.66 (2)
*V* (Å3)	8726 (2)	8777 (2)	8773 (2)
*Z*	2	2	2
radiation type	Mo Kα	Mo Kα	Mo Kα
μ (mm^–1^)	0.76	0.68	0.61
crystal size (mm)	0.24 × 0.16 × 0.09	0.21 × 0.11 × 0.10	0.17 × 0.09 × 0.09
Data Collection			
diffractometer	XtaLABSynergy R, DW system, HyPix-Arc 150	XtaLABSynergy R, DW system, HyPix-Arc 150	XtaLABSynergy R, DW system, HyPix-Arc 150
absorption correction	Gaussian	multi-scan	Gaussian
*T*_min_, *T*_max_	0.651, 1.000	0.898, 1.000	0.739, 1.000
no. of measured, independent, and observed [*I* > 2σ(*I*)] reflections	227894, 34280, 31771	212386, 32675, 28988	236299, 44455, 39035
*R*_int_	0.037	0.076	0.060
(sin θ/λ)_max_ (Å^–1^)	0.617	0.606	0.671
Refinement			
*R*[*F*^2^> 2σ(*F*^2^)], *wR*(*F*^2^), *S*	0.060, 0.157, 1.10	0.072, 0.188, 1.10	0.058, 0.157, 1.05
no. of reflections	34280	32675	44455
no. of parameters	1741	1773	1746
no. of restraints	34	1	36
H-atom treatment	H-atom parameters constrained	H-atom parameters constrained	H-atom parameters constrained
	*w* = 1/[σ^2^(*F*_o_2) + (0.0636*P*)^2^ + 40.2234*P*] where *P* = (*F*_o_^2^ + 2*F*_c_^2^)/3	*w* = 1/[σ^2^(*F*_o_^2^) + (0.0765*P*)^2^ + 47.4307*P*] where *P* = (*F*_o_^2^ + 2F_c_^2^)/3	*w* = 1/[σ^2^(*F*_o_^2^) + (0.0728*P*)^2^ + 22.8589*P*] where *P* = (*F*_o_^2^ + 2*F*_c_^2^)/3
Δ⟩_max_, Δ⟩_min_(e Å^–3^)	2.26, −1.43	2.42, −1.36	2.44, −1.06

a Computer programs: *CrysAlis PRO* 1.171.41.112a (Rigaku OD, 2021), *SHELXS97* (Sheldrick, 2008), and *SHELXL2018*/1 (Sheldrick, 2018).

The general formula of all three compounds is [MCl_3_(POSS)_3_]·2THF (M = Er^3+^, Tb^3+^, Sm^3+^), and they all contain an octahedrally coordinated metal cation (Er^3+^, Tb^3+^, and Sm^3+^ for **3**, **4** and **5**, respectively). Erbium (**3**) and samarium (**5**) structures are strictly isomorphous because they differ only in the metal atom in the molecule’s center.

**Figure 1:**
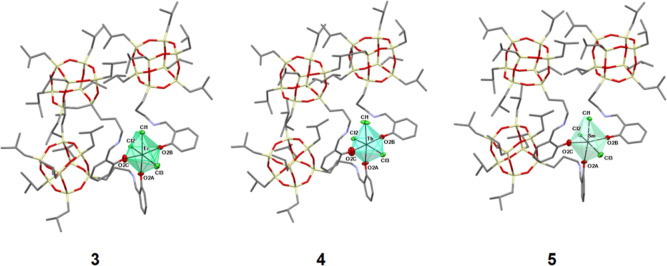
Molecular structures of **3**, **4**, and **5**. Hydrogen atoms are omitted for clarity. For the sake of clarity, the image is presented in stick mode, omitting the thermal ellipsoids.

Compounds **3** and **5** consist of a six-coordinated metal cation bound to three oxygen atoms and three chlorine anions (Figure 1). The oxygen atoms belong to the three phenolate groups of the three POSS ligands, while the chlorine atoms are derived from the lanthanide(III) chloride salts used in the synthesis. Those molecules have a distorted octahedral geometry and are meridional (mer) isomers.

**Figure 2:**
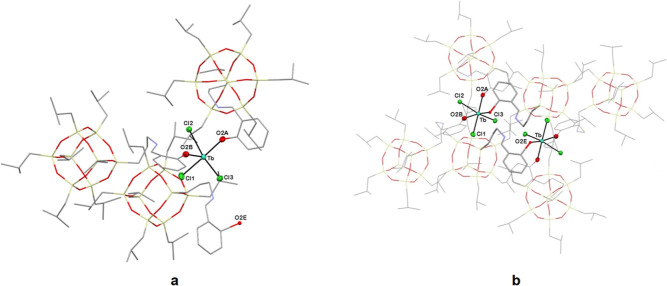
Asymmetric unit (**a**) and dimer molecule (**b**) of **4**. Hydrogen atoms are omitted for clarity. For the sake of clarity, the image is presented in stick mode, omitting the thermal ellipsoids.

However, surprisingly, the terbium compound is only 65% identical to compounds **3** and **5**. In the crystal structure of **4**, there are two different molecules. One of them is a monomer with the same structure as the erbium and samarium species. On the other hand, 35% is a dimer, in which there are two metallic centers. In this case, the Tb^3+^ cation is bonded to only two oxygen atoms of POSS ligands. The imine arm of the third cage is bent in the opposite direction around the Si10C–C10C bond. As a result, the torsion angle between O13C–Si1C–C10C–C9C/C9E changes from 81.21(2) to −75.73(2)^o^, and the oxygen atom of the third POSS cage is coordinated to the second lanthanide cation so that both metal centers have octahedral geometry (Figure 2). The dimer molecule of the terbium compound was created as a result of a different arrangement in the space of one of the imine arms of the POSS and is probably also energetically beneficial.

In the crystal structures of **3–5**, apart from the lanthanide complexes, there are also two non-coordinated molecules of tetrahydrofuran, which was used as a solvent during synthesis. Selected bond lengths and angles for **3**, **4**, and **5** are presented in Table 2.

**Table 2:** Selected Bond Lengths (Å) and Angles (°) for **3**, **4**, and **5**^a^

**3**	Er–O2A	2.230 (3)	O2C–Er–O2B	171.17 (14)	O2B–Er–Cl3	88.99 (9)
	Er–O2B	2.239 (3)	O2A–Er–O2B	96.82 (11)	Cl1–Er–Cl3	95.78 (10)
	Er–O2C	2.161 (3)	O2C–Er–Cl1	85.48 (12)	O2C–Er–Cl2	96.81 (10)
	Er–Cl1	2.6155 (14)	O2A–Er–Cl1	175.71 (12)	O2A–Er–Cl2	81.64 (8)
	Er–Cl2	2.6490 (13)	O2B–Er–Cl1	85.93 (10)	O2B–Er–Cl2	81.96 (9)
	Er–Cl3	2.6163 (16)	O2C–Er–Cl3	93.93 (11)	Cl1–Er–Cl2	95.52 (7)
	O2C–Er–O2A	91.64 (14)	O2A–Er–Cl3	87.58 (11)	Cl3–Er–Cl2	164.97 (7)
**4**	Tb–O2A	2.266 (4)	O2E^i^–Tb–O2B	167.5 (3)	O2A–Tb–Cl3	84.4 (3)
	Tb–O2B	2.271 (4)	O2C–Tb–O2B	177.3 (3)	O2B–Tb–Cl3	88.49 (16)
	Tb–O2C	2.253 (11)	O2A–Tb–O2B	97.99 (14)	Cl1–Tb–Cl3	100.4 (2)
	Tb–O2E^i^	2.156 (17)	O2E^i^–Tb–Cl1	81.6 (3)	O2E^i^–Tb–Cl2	96.2 (4)
	Tb–Cl1	2.6457 (16)	O2C–Tb–Cl1	92.2 (4)	O2C–Tb–Cl2	100.8 (3)
	Tb–Cl2	2.6930 (15)	O2A–Tb–Cl1	173.71 (12)	O2A–Tb–Cl2	81.18 (10)
	Tb–Cl3	2.678 (4)	O2B–Tb–Cl1	86.30 (10)	O2B–Tb–Cl2	81.50 (10)
	O2E^i^–Tb–O2A	93.8 (4)	O2E^i^–Tb–Cl3	96.9 (4)	Cl1–Tb–Cl2	94.99 (5)
	O2C–Tb–O2A	83.7 (4)	O2C–Tb–Cl3	89.6 (3)	Cl3–Tb–Cl2	161.04 (15)
**5**	Sm–O2A	2.309 (2)	O2C–Sm–O2B	169.93 (10)	O2B–Sm–Cl3	87.61 (7)
	Sm–O2B	2.321 (2)	O2A–Sm–O2B	99.86 (9)	Cl1–Sm–Cl3	98.47 (6)
	Sm–O2C	2.245 (3)	O2C–Sm–Cl1	84.47 (9)	O2C–Sm–Cl2	99.06 (7)
	Sm–Cl1	2.6806 (11)	O2A–Sm–Cl1	173.54 (8)	O2A–Sm–Cl2	80.50 (6)
	Sm–Cl2	2.7283 (11)	O2B–Sm–Cl1	85.59 (7)	O2B–Sm–Cl2	80.54 (7)
	Sm–Cl3	2.7042 (12)	O2C–Sm–Cl3	95.47 (8)	Cl1–Sm–Cl2	97.10 (5)
	O2C–Sm–O2A	89.97 (10)	O2A–Sm–Cl3	85.31 (7)	Cl3–Sm–Cl2	159.61 (3)

a Symmetry code (s): (i) −*x* + 1, −*y* + 1, −*z* + 1.

The obtained compounds can be compared with molecules containing similar fragments, in which the lanthanide cation is coordinated with the chlorine atoms and the oxygen atoms of the phenolate group. The lengths of M–O bonds with the oxygen atoms of the phenolate groups of POSS ligands in compounds **3**, **4**, and **5** are similar. Their values are given in the following ranges: 2.161 (3)–2.239 (3) Å for **3**, 2.253 (11)–2.271 (4) Å for **4**, 2.245 (3)–2.321 (2) Å for **5**. Slight differences in the lengths of the M–O bonds in compounds **3**, **4**, and **5** result from the different lengths of the ionic radii of the lanthanide cations. These values correspond to the length of the M–O bonds found in the compounds Eu, Dy, and Er described by Huang et al.^44^ and Tb, Dy, Ho, and Er reported by Li et al.,^45^ which contain M–O–Ph fragments.

A similar relation occurs for the length of M–Cl bonds in compounds **3**, **4**, and **5**, the values of which are in the following ranges: **3**, 2.6155 (14)–2.6490 (13) Å; **4**, 2.6457 (16)–2.6930 (15) Å; and **5**, 2.6806 (11)–2.7283 (11) Å. These values also correspond to the literature data. In this case, erbium, terbium, and samarium compounds were compared with octahedral lanthanide compounds described by Li et al.,^45^ in which three chlorine anions coordinate to the metal cation.

**Figure 3:**
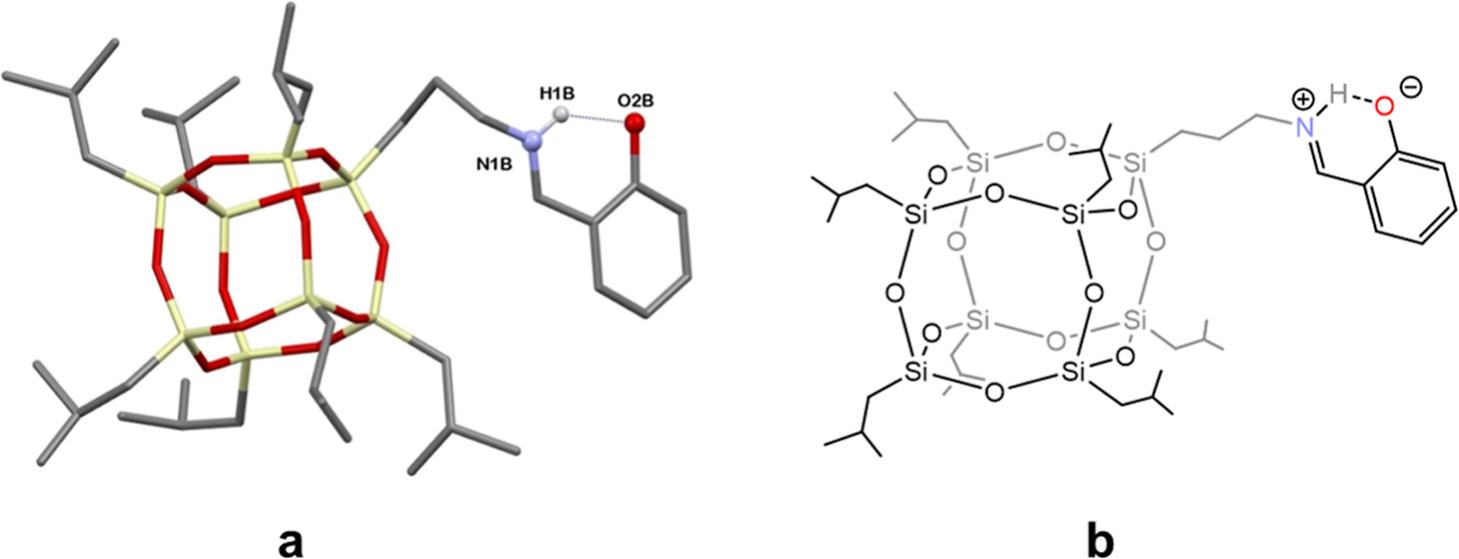
Fragment of the molecular structure of **3** showing the POSS ligand with marked hydrogen bond (**a**); the structural formula of the POSS ligand (**b**).

Intramolecular hydrogen bonds additionally stabilize the structures of **3–5** coordination entities. These bonds occur in all silsesquioxane ligands between the oxygen atoms of the phenolate groups and the NH^+^ moiety (Figure 3). Due to the tautomerism that occurs on the imine arms of the POSS, these moieties are zwitterions. As a result, the hydroxyl groups of the salicylic fragment of the molecule are deprotonated, while the positive charge is on the nitrogen atom.

### Optical Properties of **3–5**

**Figure 4:**
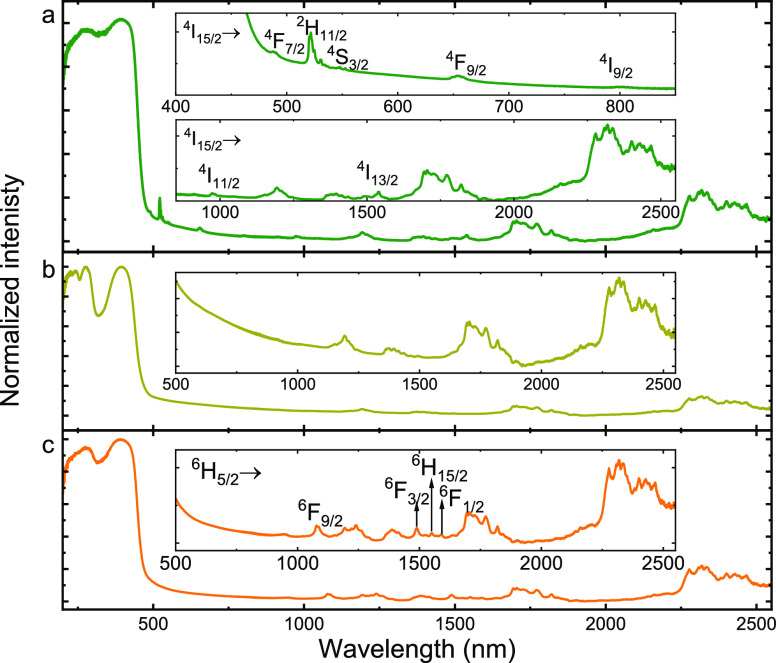
Diffuse reflectance spectra of **3** (a), **4** (b), and **5** (c) samples recorded at room temperature.

The diffuse reflectance spectra of **3–5** compounds measured at 300 K are presented in Figure 4.

It can be seen that all samples reveal a broad band in the range of 200–490 nm, which can be attributed to material absorption. The mentioned band is so wide and intense that it covers the visible range in which the absorption transitions of embedded lanthanides usually occur. Because of its electronic structure, only narrow bands from Er^3+^(**3**) ions can be observed there.^46^ In the case of the NIR range, the absorption transitions characteristic of Er^3+^(**3**) and Sm^3+^(**5**) ions in the respective analogues have been identified (see Figure 4, insets).^46,47^

The absorption spectra were recalculated to determine the energy band gap (*E*_g_) of the **3–5** samples using Kubelka–Munk formula^48^
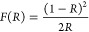
1where *R* means reflectance. The *E*_g_ of analyzed materials was calculated to be 2.7 eV regardless of the lanthanide contained in the crystal structure (see Figure S12).

**Figure 5:**
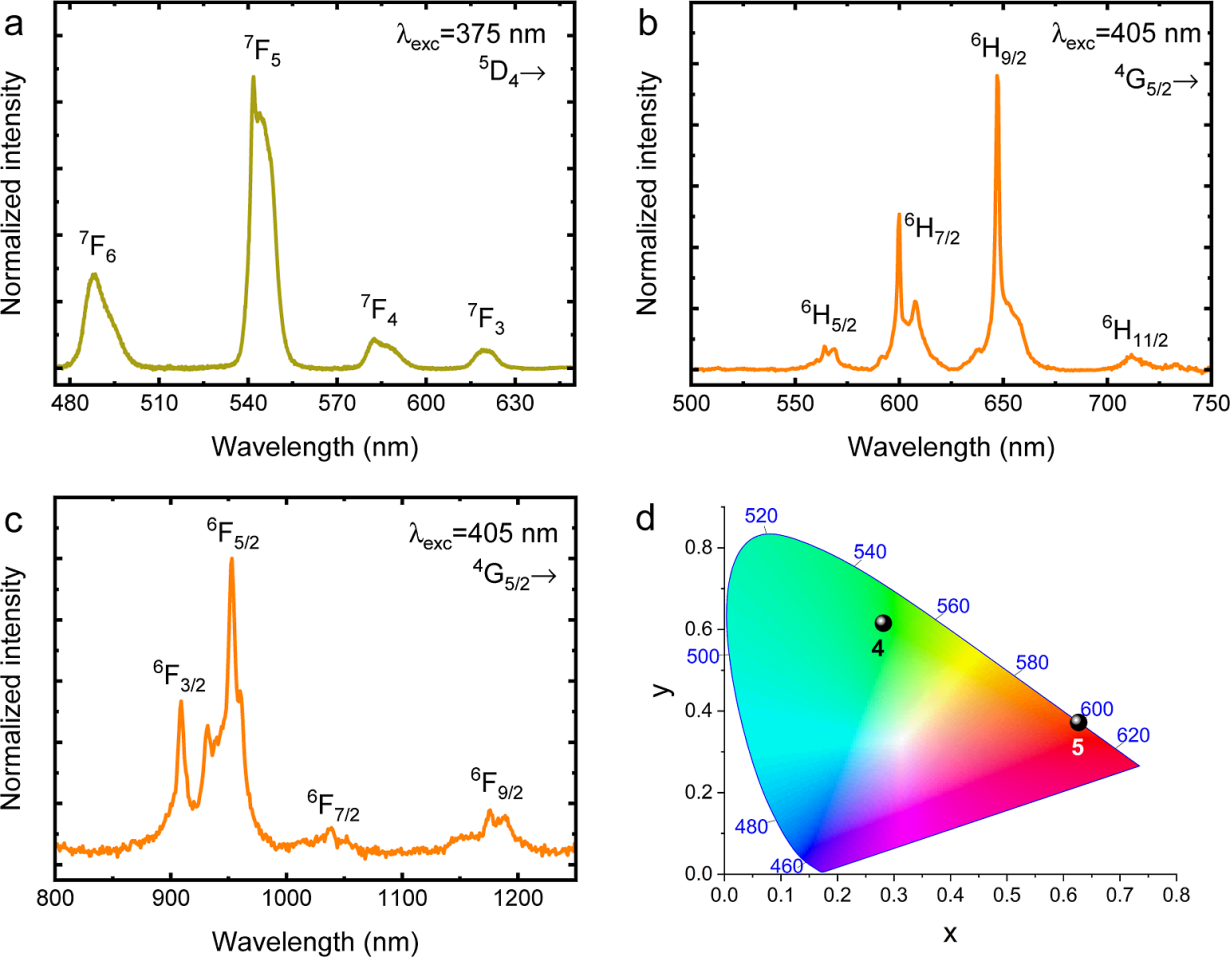
Emission spectra of **4** (a) and **5** (b,c) samples recorded at room temperature and their chromatic coordinates (d).

In order to characterize the optical properties of the investigated materials, their emission spectra were recorded. The measurements were performed using the excitation lines of 375 and 405 nm for the sample containing Tb^3+^(**4**) and Sm^3+^(**5**) ions, respectively, for the direct population of higher levels of these lanthanides (Figure 5).

During the research, it was found that **3** and **4** excited by the 405 nm line corresponding to the most intense absorption band did not generate any spectral response. This proves the lack of energy transfer between the lanthanide ions and the ligand in these species. It is worth mentioning that none of the excitation lines typical of the Er^3+^ (**3**) produced no luminescence from this rare earth ion, in neither the visible (VIS) range nor the infrared (NIR) range, probably due to strong non-radiative processes.^49^ However, further detailed analysis is needed to investigate the nature of this luminescence quenching thoroughly. Figure 5a demonstrates the emission spectrum of **4** recorded under 375 nm excitation at 300 K. It can be seen that the sample shows four narrow spectral lines in the range of 480–630 nm, characteristic for Tb^3+^ ions. Bands with a maximum at 488, 542, 584, and 620 nm can be assigned to transitions from the ^5^D_4_ emission level to ^7^F_6_, ^7^F_5_, ^7^F_4_, and ^7^F_3_ states, respectively.^50,51^Figure 5b,c shows the emission spectra of **5** compound measured upon 405 nm excitation at 300 K. The sample exhibits typical Sm^3+^ transitions in both the visible and infrared spectral ranges. Bands centered at 564, 600, 647, and 711 nm in VIS and 909, 953, 1037, and 1175 nm in NIR were attributed to transitions from the ^4^G_5/2_ emission level to ^6^H_5/2_, ^6^H_7/2_, ^6^H_9/2_, and ^6^H_11/2_ and ^6^F_3/2_, ^6^F_5/2_, ^6^F_7/2_, and ^6^F_9/2_ states, respectively.^52,53^ The luminescence decay profiles for the dominant Tb^3+^ (**4**) (^5^D_4_ → ^7^F_5_) and Sm^3+^ (**5**) (^4^G_5/2_ → ^6^H_9/2_) bands were recorded and are plotted in Figure S13. The curves were well fitted by a biexponential function indicating the presence of some non-radiative processes. The average lifetimes for the Tb^3+^ and Sm^3+^ ions calculated using eq 2 were equal to 0.68 ms and 13.7 μs, respectively
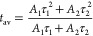
2Here, *A*_1_ and *A*_2_ are the fitting constants, and τ_*i*_ (*i* = 1, 2) means the decay time of the *i* component.

In order to visualize the color of the registered luminescence of **4** and **5** coordination entities, their chromatic coordinates were determined and are shown in Figure 5d. It turned out that their emission color is located in the yellowish-green and reddish-orange regions, respectively.

### Magnetic Properties of **3–5**

Because of their large and anisotropic magnetic moment, lanthanide cations and their coordination entities are of constant interest in molecular magnetism.^54−56^ The anisotropy of magnetic susceptibility at low temperatures is primarily determined by the anisotropy of the *g*-tensor of the ground electronic level and a few low-lying crystal field levels of the lanthanide ions. With increasing temperature, the crystal field levels at higher energy are progressively occupied, and magnetic anisotropy decreases rapidly, although still very noticeable at room temperature and above. However, interpreting the magnetic data of Ln^3+^ compounds is continuously challenging because of the large orbital contribution of these ions. Another issue for these, usually of low symmetry, species is the disentanglement of the single-ion magnetic anisotropy from the exchange contributions in the analysis of the temperature dependence of the magnetic susceptibility.

**Figure 6:**
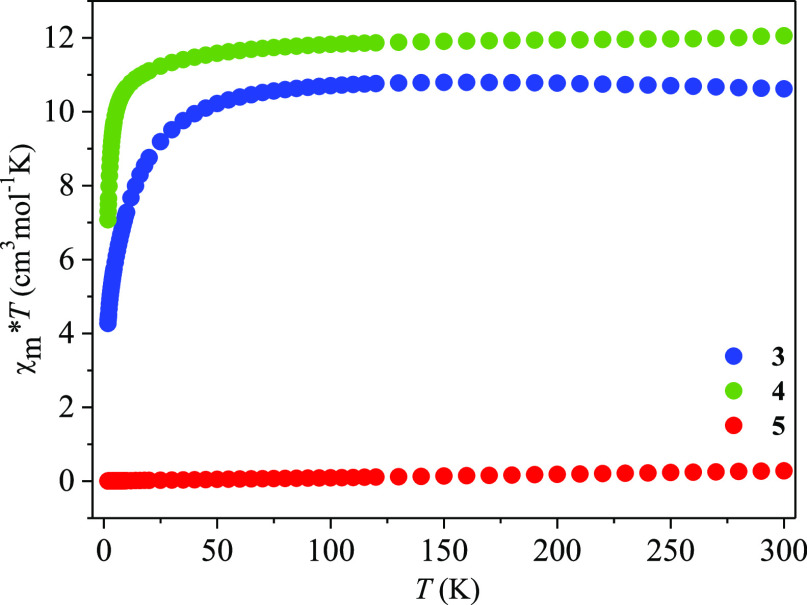
Temperature dependence of experimental χ_m_*T* for **3–5** (χ_m_ per Ln^3+^ ion).

Direct current (dc) magnetic susceptibility measurements were carried out on a polycrystalline sample of **3–5** lanthanide complexes between 1.8 and 300 K with an applied magnetic field of 0.5 Tesla. Plots of χ_m_*T* product versus *T* (χ_m_ is the molar magnetic susceptibility per Ln^3+^ ion) for **3–5** are given in Figure 6.

The experimentally determined values of χ_m_*T* of investigated coordination entities at room temperature were compared with the theoretical values of χ_m_*T* calculated by the equation , where *N* is the Avogadro constant, β is the Bohr magneton, *k* is Boltzman’s constant, and *g*_Ln_ is the *g* factor of the ground *J* terms of Ln^3+^ expressed as *g*_Ln_ = 3/2 + *S*(*S* + 1) – *L*(*L* + 1)/2*J*(*J* + 1).^57^ At 300 K, the χ_m_*T* products of 10.62 cm^3^ K mol^–1^ (**3**) and 12.06 cm^3^ K mol^–1^ (**4**) are close to the expected values for single non-interacting Er^3+^ (11.48 cm^3^ K mol^–1^, ^4^*I*_15/2_, *S* = 3/2, *L* = 6, *J* = 15/2, *g* = 6/5) and Tb^3+^ (11.80 cm^3^ K mol^–1^: ^7^*F*_6_, *S* = 3, *L* = 3, *J* = 6, *g* = 3/2) ions, respectively. The values are within the range previously found for mononuclear Er^3+^ and Tb^3+^ compounds.^58−61^ Although there are two different molecules in the crystal structure of the terbium compound, monomer and dimer, magnetic studies correspond to only one metal center. Upon cooling, the χ_m_*T* for both compounds remains relatively constant down to 100 K, below which it begins a steeper reduction to 4.27 cm^3^ mol^–1^ K (**3**) and 7.08 cm^3^ mol^–1^ K (**4**) at 1.8 K. The gradual decrease of χ_m_*T* upon cooling can be explained by a progressive depopulation of excited Stark sublevels because of the ligand field effects. This suggests the presence of significant magnetic anisotropy characteristics for lanthanide(III)-containing coordination entities.^61−66^ Such behavior of **3** and **4** can also be attributed to weak intermolecular interactions. The plot of χ_m_^–1^ versus T obeys the Curie–Weiss law in the whole temperature range with a negative Weiss constant θ = −3.52 K (**3**) and −1.47 K (**4**). The negative values of the Weiss temperatures, in combination with the changing tendency of the temperature dependence of *χ*_m_*T* for both complexes, lead to a conclusion that the magnetic interaction is weak antiferromagnetic. However, the great intermolecular metal–metal separation in these mononuclear coordination entities could not favor the exchange coupling. Unfortunately, the quantitative description of the magnetic properties of lanthanide(III) species is cumbersome due to the ligand-field effect and spin–orbit coupling of the Ln^3+^ cation.^67^ Magnetization studies verified the nature of the ground state of **3** and **4** at 2–8 K in the field range of 0–7 T (Figures S14 and S15, respectively). The *M* versus *H* curves for both complexes show a sharp increase in magnetization at a low field limit with a linear response to the magnetic moment upon increasing the magnetic field without any saturation. The values of the magnetization at 2 K and 7 T are 5.63 μ_B_ (**3**) and 5.53 μ_B_ (**4**), respectively. They are well below the expected value for the saturation of the magnetization of free, non-interacting Er^3+^ and Tb^3+^ ions, which is 9 μ_B_ (considering *J* = 15/2 and *J* = 6 ground state, respectively). Non-saturation of magnetization at a high field limit (at 2 K) might arise from the intermolecular interactions, the magnetic anisotropy, and significant crystal field effects associated with both complexes. As discussed above, taking into account the quite long intermolecular distance between the closest Ln···Ln centers, it seems unlikely that the intermolecular interactions will have a substantial impact on the magnetism. The presence of single-ion magnetic anisotropy is also confirmed by the non-superimposable nature of the magnetization curves at higher temperatures (Figures S14 and S15, respectively). Due to the large anisotropy,^60,61^ some terbium(III) or erbium(III) complexes show SMM behavior. Upon application of a static dc field of 0.1 T, both in-phase (χ′) and out-of-phase (χ″) susceptibilities show no frequency dependence peaks in the 1.8–25 K temperature range for an oscillating field range of 1 to 900 Hz for complexes **3** and **4**, which exclude the presence of magnetic ordering or slow paramagnetic relaxation. This suggests that the anisotropy around lanthanide(III) ions is not large enough in both complexes for the requirement of SMM, which may probably be quenched by the very weak intermolecular interaction. The other important reason is the poorly axial symmetry of the coordination geometry of the Tb^3+^ and Er^3+^ ions.

The ^6^H ground term for the free Sm^3+^ ion is split into six levels by spin–orbit coupling, and the spin–orbit coupling parameter is 200 cm^–1^. This means that the crystal-field effect and the possible thermal population of the higher states should be assessed for the samarium(III) coordination entity. As shown in Figure 6, the plot of χ_m_*T* versus *T* for **5** is nearly linear over the whole temperature range and similar to that reported for mononuclear samarium(III) compounds.^68−72^ Therefore, the relationship of 1/χ_m_ versus *T* does not obey Curie law or Curie–Weiss law. The value of χ_m_*T* is equal to 0.279 cm^3^ K mol^–1^ at room temperature and decreases rapidly with the temperature to a value of 0.03 cm^3^ K mol^–1^ at 1.8 K, which is lower than the value of 0.089 cm^3^ K mol^–1^ predicted by theory. This difference may be because the ^6^H_5/2_ ground state of Sm^3+^ is split into three Kramers doublets.^57^

## Conclusions

In this publication, we presented the use of the mono-substituted imine-POSS **2**, which was obtained in the reaction of 3-aminopropylheptaisobutyl-POSS, as a ligand for selected lanthanide ions, such as erbium (**3**), terbium (**4**), and samarium (**5**) of the formula [MCl_3_(POSS)_3_]·2THF (M = Er^3+^, Tb^3+^, Sm^3+^). The structure of the ligand and the coordination compounds was fully confirmed in solution and solid states. The X-ray analysis of single crystals showed that the erbium and samarium complexes are isomorphous because they differ only in the metal cation in the molecule’s center. However, surprisingly, the terbium compound is only 65% identical to compounds **3** and **5**. In the crystal structure of **4**, there are two different molecules. One of them is a monomer with the same structure as the erbium and samarium species. On the other hand, 35% is a dimer, in which there are two metallic centers. Measurements of the optical properties of **3–5** coordination entities have shown that apart from the narrow absorption transitions characteristic of lanthanides, a wide broad material band corresponding to the matrix’s absorption, covering the spectral range up to 490 nm, is also observed. These measurements made it possible to determine the energy band gap of the studied compounds equal to 2.7 eV, regardless of the lanthanide contained in their crystal structure. Direct excitation of rare earth ions led to the appearance of luminescence with spectral characteristics typical for the investigated lanthanides. The exception was the analogue containing Er^3+^ ions, whose luminescence was quenched through non-radiative processes. In turn, **5** shows behavior characteristics for the well-isolated mononuclear system, whereas the magnetic properties of **3** and **4** are dominated by the significant orbital contributions and the crystal field effect on the lanthanide(III) site, masking the magnetic interaction between the paramagnetic centers.

## Supporting Information Available

NMR (^1^H, ^13^C, ^29^Si), FT-IR spectra, additional optical and magnetic properties, and crystallographic refinement details (PDF)
