# Supplementary material for: Synthesis, Crystal Structures, and Optical and Magnetic Properties of Samarium, Terbium, and Erbium Coordination Entities Containing Mono-Substituted Imine Silsesquioxane Ligands
Source: Inorg Chem. 2023 Jan 30;62(6):2913–23. doi: 10.1021/acs.inorgchem.2c04371 (PMC9930112; doi:10.1021/acs.inorgchem.2c04371)
Supplement: Supplementary file 1 — ic2c04371_si_001.pdf [file ic2c04371_si_001.pdf]

Supplementary Materials

Synthesis, Crystal Structures, and Optical and Magnetic Properties of Samarium, Terbium, and Erbium Coordination Entities Containing Mono-Substituted Imine Silsesquioxane Ligands

Patrycja Wytrych[a], Józef Utko[a], Mariusz Stefanski[b], Julia Kłak[a], Tadeusz Lis[a] and Łukasz John[a,]*

[a]Faculty of Chemistry, University of Wrocław, 14 F. Joliot-Curie, 50-383 Wrocław, Poland

[b]Institute of Low Temperature and Structure Research, Polish Academy of Sciences, 2 Okólna,50-422 Wrocław, Poland

Corresponding author: Łukasz John, e-mail: lukasz.john@uwr.edu.pl

Table of Contents

1. **Characterization of 1**

Figure S1. ^{1}H NMR (500 MHz, $CDCl_3$, 300 K) spectrum of **1**……………………………S2

Figure S2. ^{13}C NMR (500 MHz, $CDCl_3$, 300 K) spectrum of **1**…………………………...S2

Figure S3. ^{29}Si NMR (500 MHz, $CDCl_3$, 300 K) spectrum of **1**…………………………..…S3

Figure S4. FT-IR spectrum (KBr pellet) of **1**……………………………………………….…..S3

2. **Characterization of 2**

Figure S5. ^{1}H NMR (500 MHz, $CDCl_3$, 300 K) spectrum of **2**…………………………….....S4

Figure S6. ^{13}C NMR (500 MHz, $CDCl_3$, 300 K) spectrum of **2**……………………………..…..S4

Figure S7. ^{29}Si NMR (500 MHz, $CDCl_3$, 300 K) spectrum of **2**…………………………….….S5

Figure S8. FT-IR spectrum (KBr pellet) of **2**…………………………………………………...S5

Characterization of 3

Figure S9. FT-IR spectrum (KBr pellet) of **3**…………………………………………………...S6

3. **Characterization of 4**

Figure S10. FT-IR spectrum (KBr pellet) of **4**……………………………………………….S6

4. **Characterization of 5**

Figure S11. FT-IR spectrum (KBr pellet) of **5**……………………………………………......S7

5. **Optical properties of 3-5**

Figure S12. The energy band gap of **3-5** species……………………………………………..S7

Figure S13. The luminescence decay profiles of **4** and **5** species………………...............….S8

6. **Magnetic properties of 3 and 4**

Figure S14. Field dependence of the magnetization for **3** (*M* per Er^{3+} ion) at 2−8 K……...…S8

Figure S15. Field dependence of the magnetization for **4** (*M* per Tb^{3+} ion) at 2−8 K……..…S9

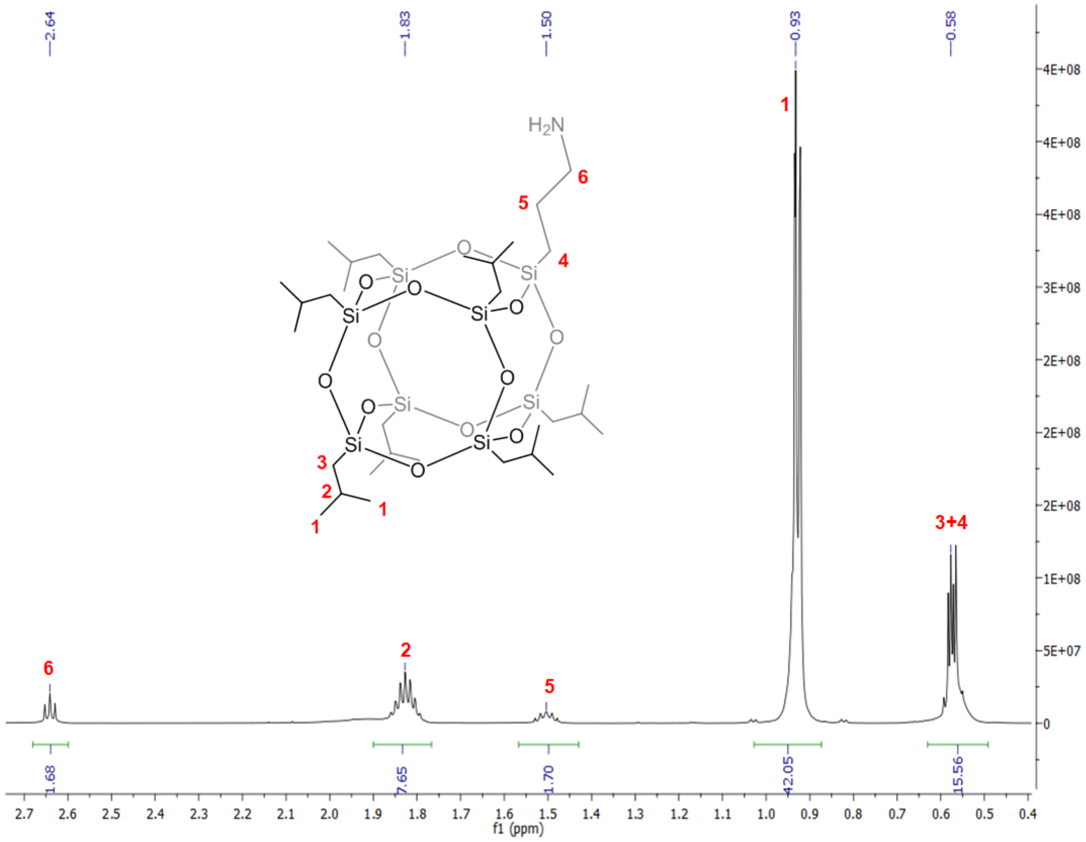

Figure S1. ^{1}H NMR spectrum of **1**.

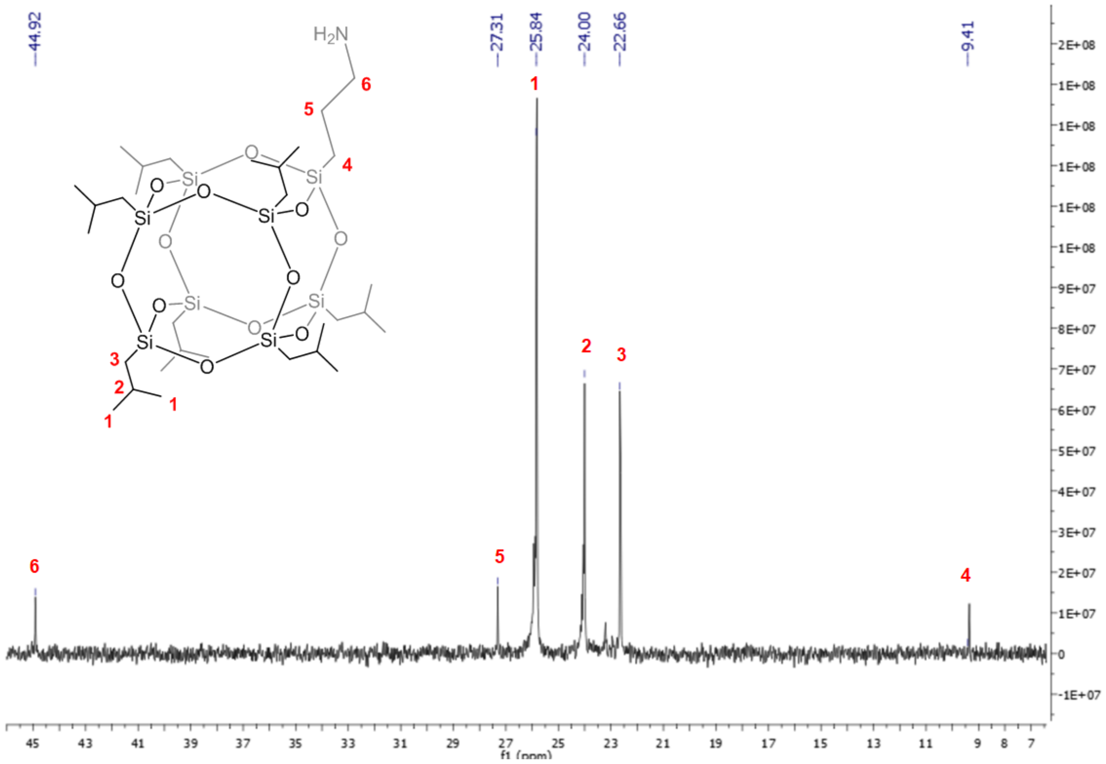

Figure S2. ^{13}C NMR spectrum of **1**.

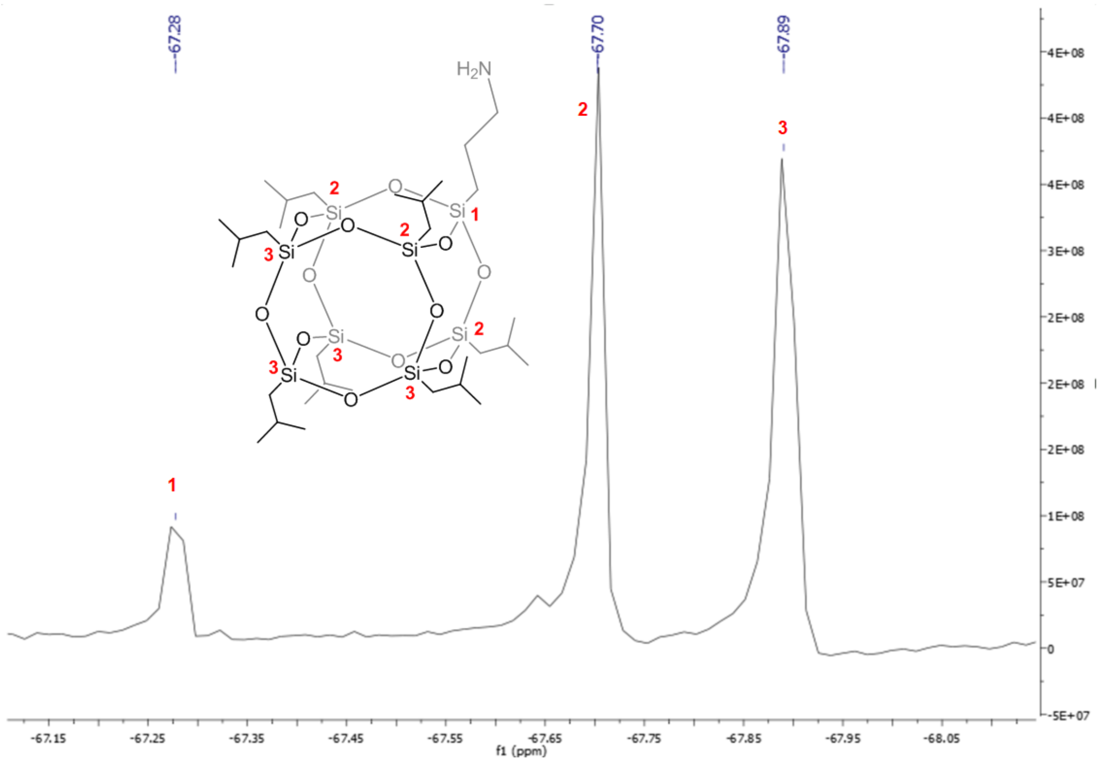

Figure S3. ^{29}Si NMR spectrum of **1**.

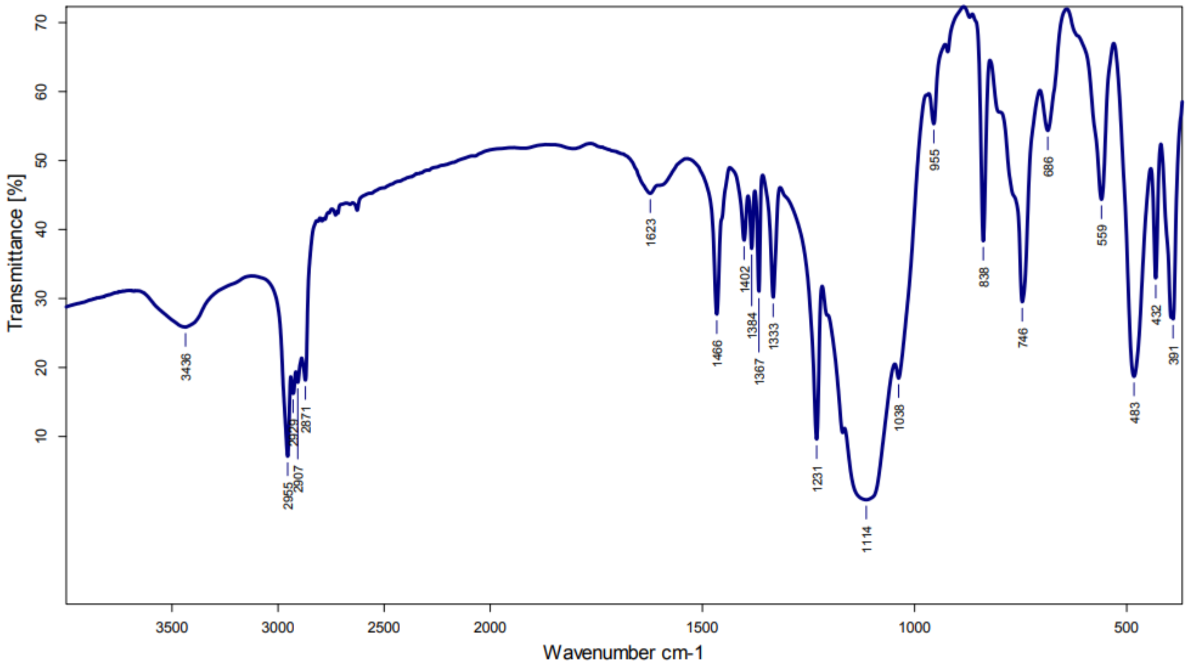

Figure S4. FT-IR spectrum of **1**.

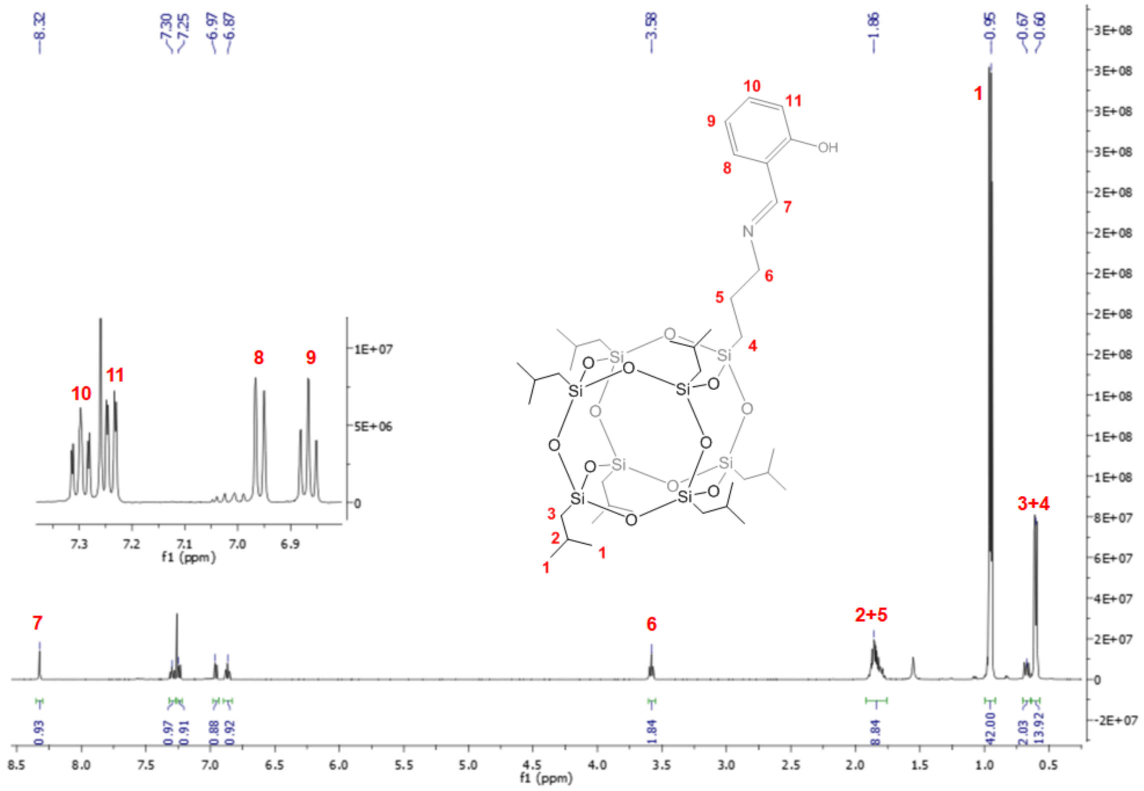

Figure S5. ^{1}H NMR spectrum of **2**.

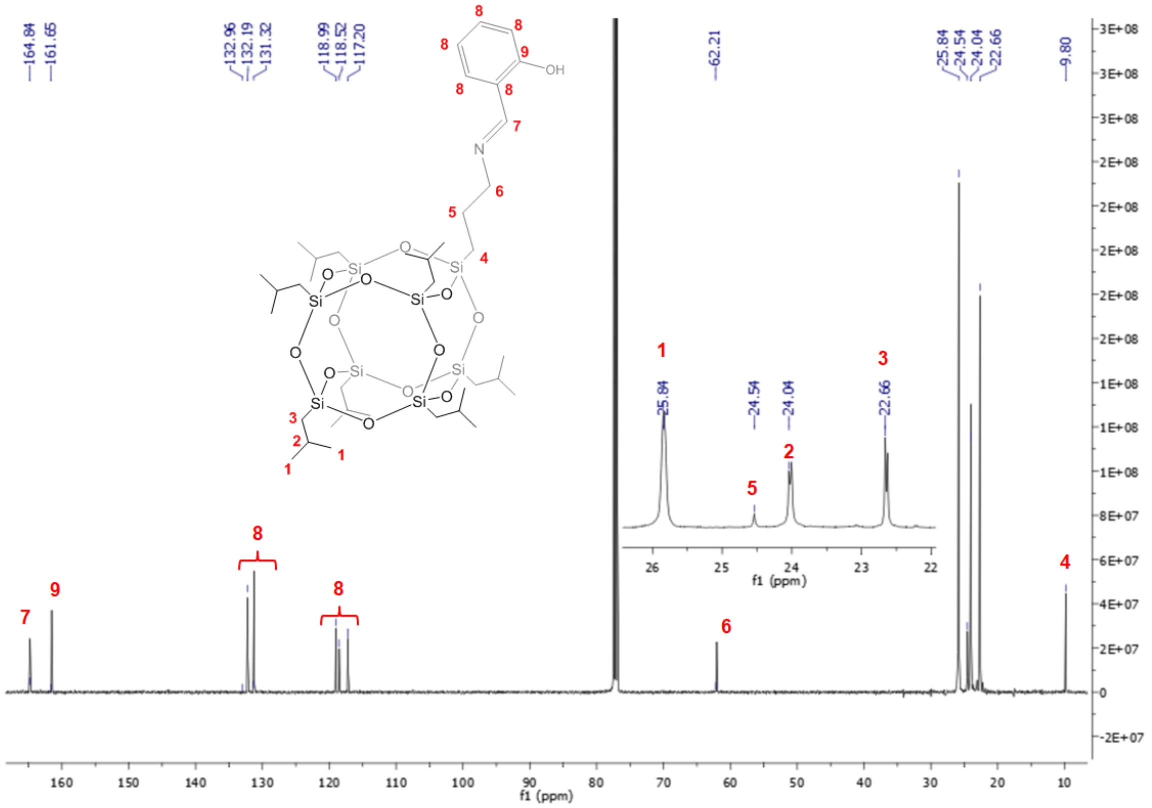

Figure S6. ^{13}C NMR spectrum of **2**.

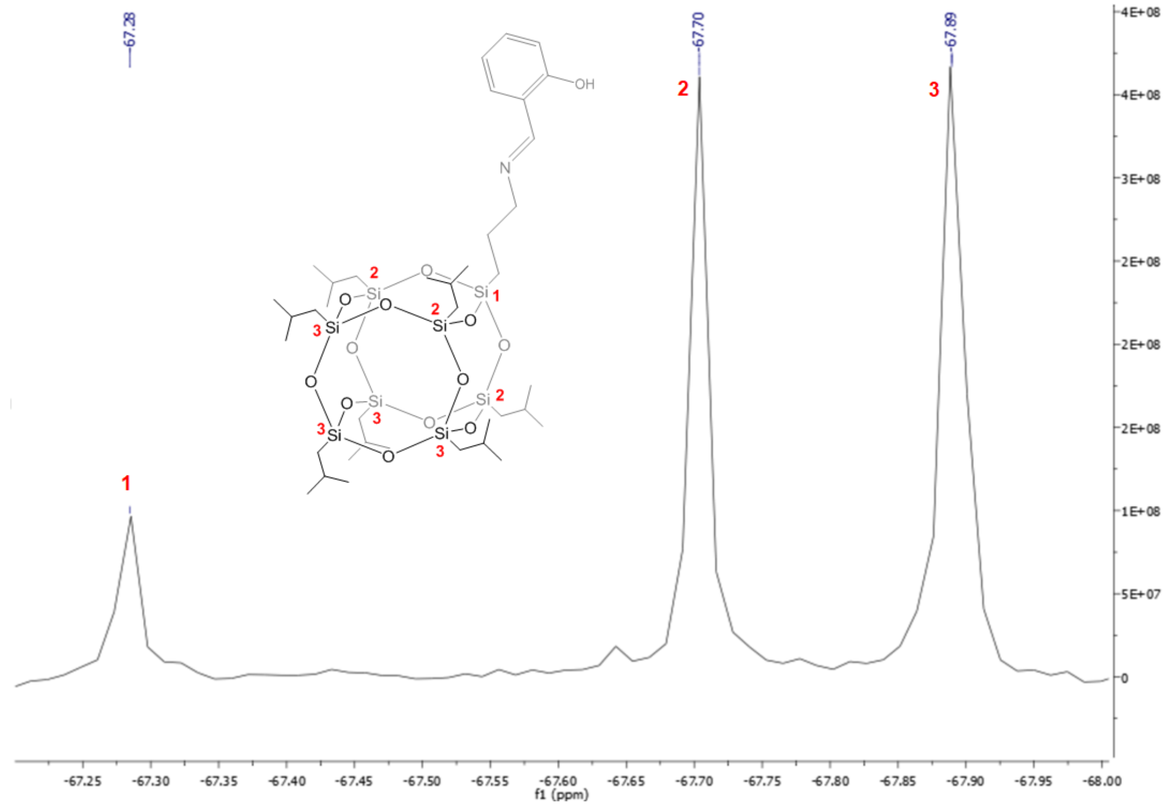

Figure S7. ^{29}Si NMR spectrum of **2**.

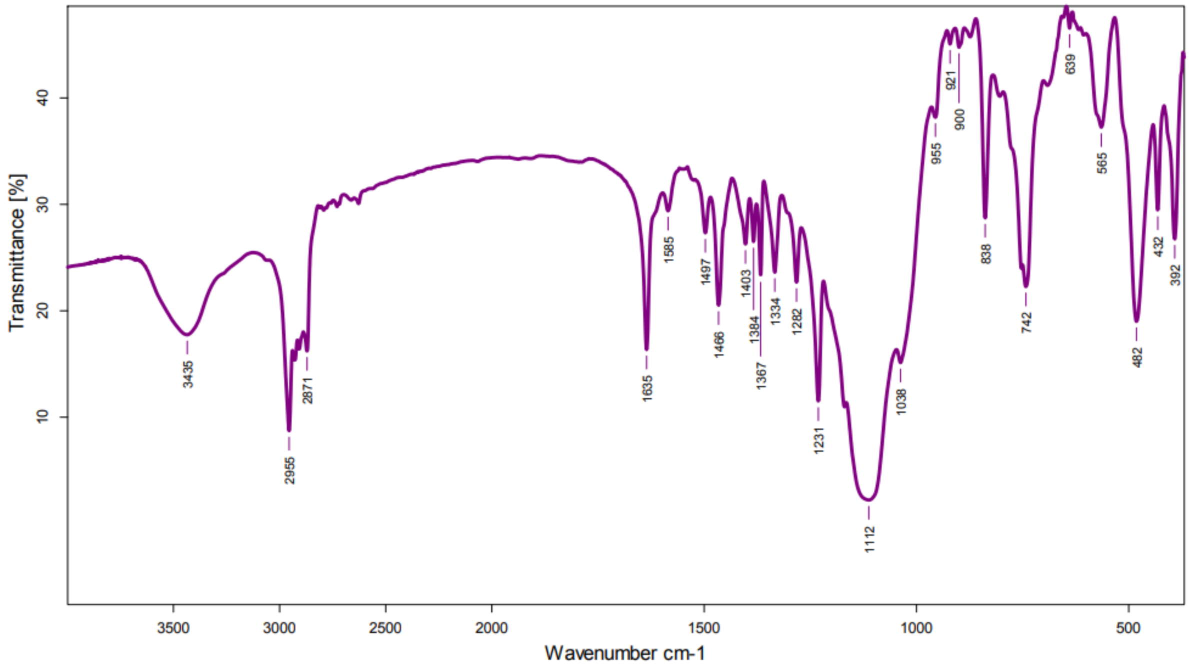

Figure S8. FT-IR spectrum of **2**.

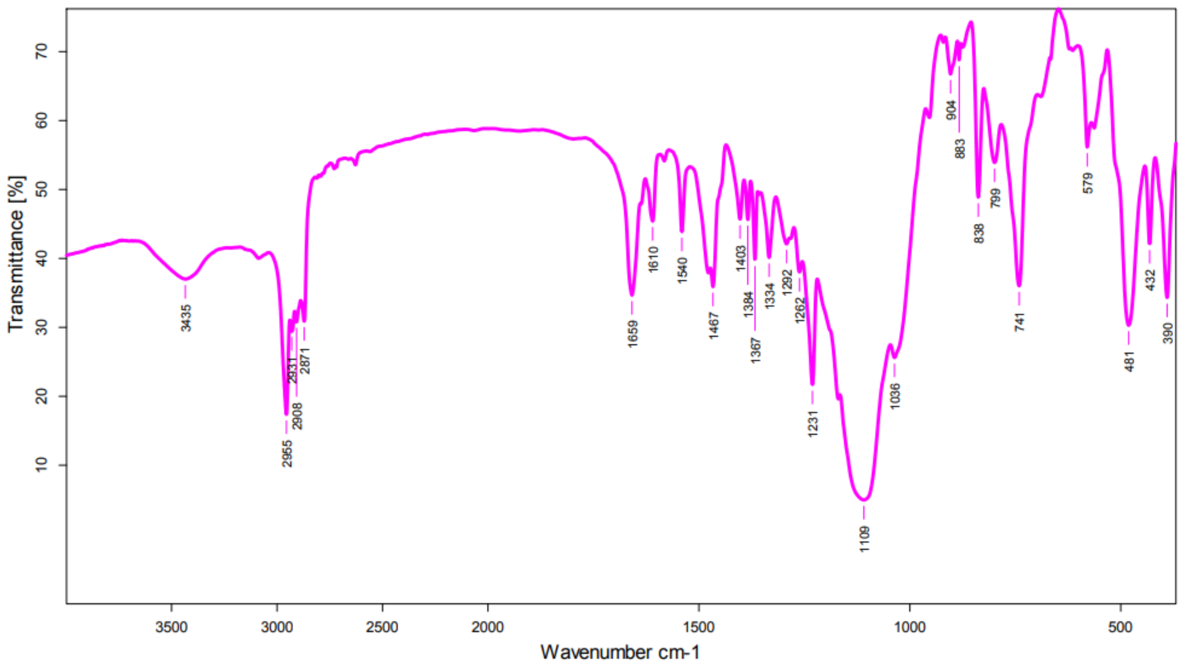

Figure S9. FT-IR spectrum of **3**.

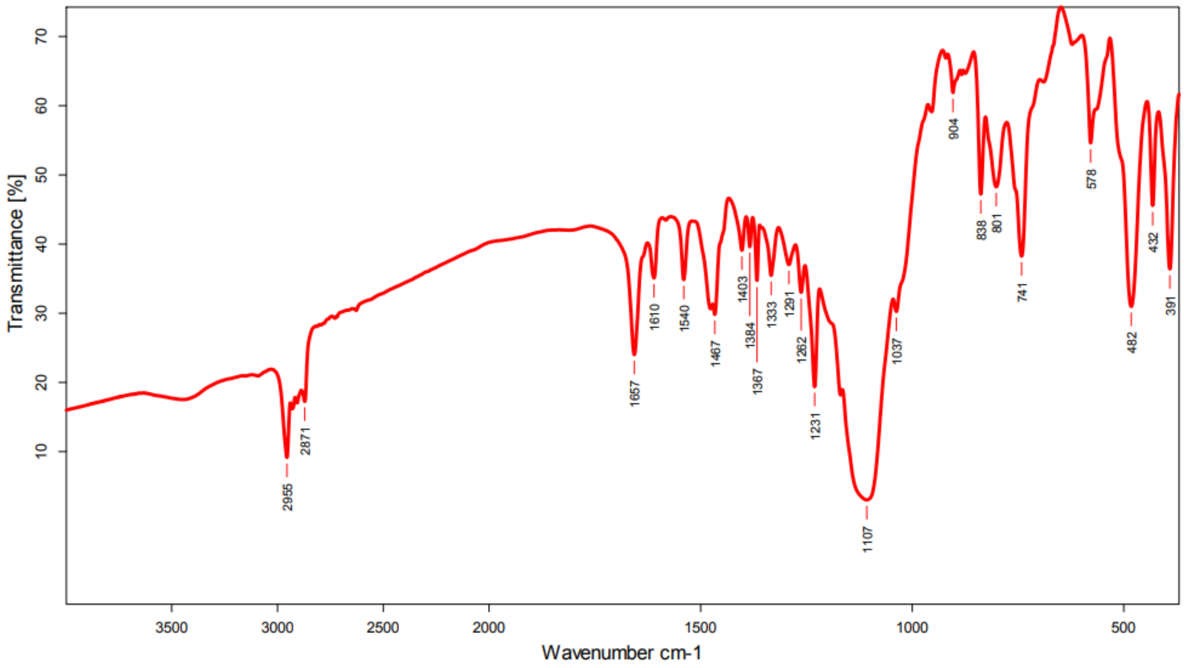

Figure S10. FT-IR spectrum of **4**.

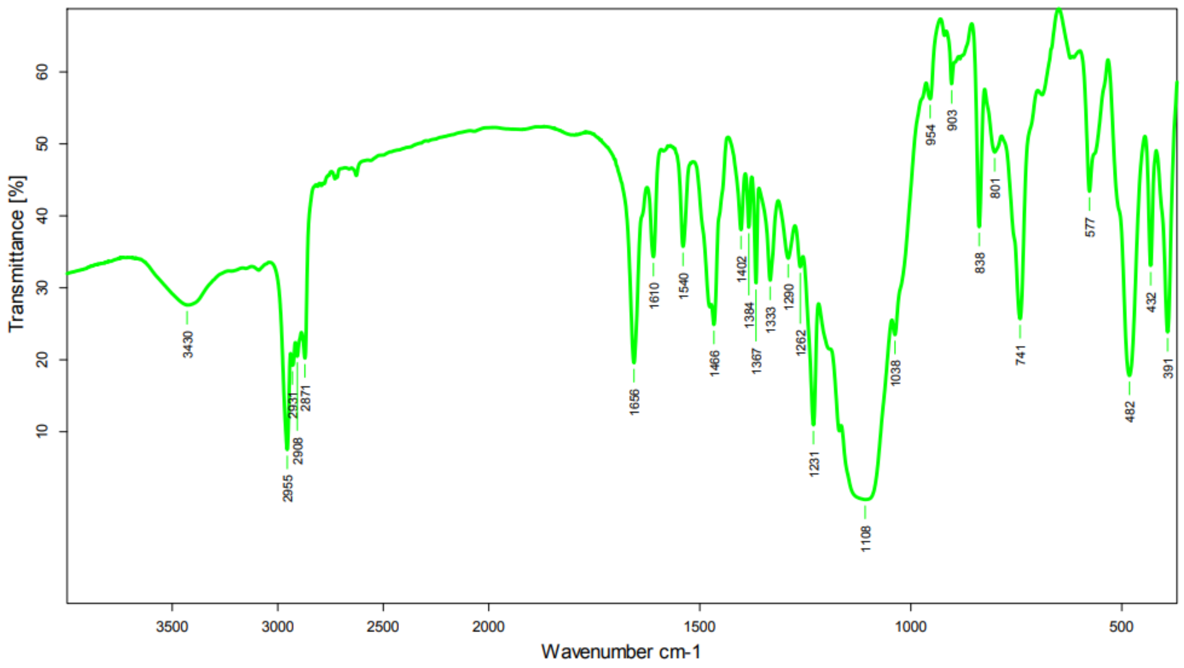

Figure S11. FT-IR spectrum of **5**.

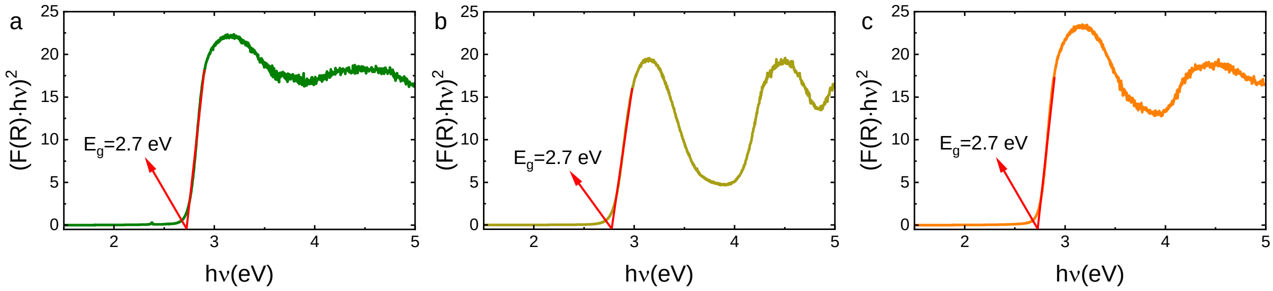

Figure S12. The energy band gap of **3** (a), **4** (b) and **5** (c) species calculated using Kubelka-Munk formula.

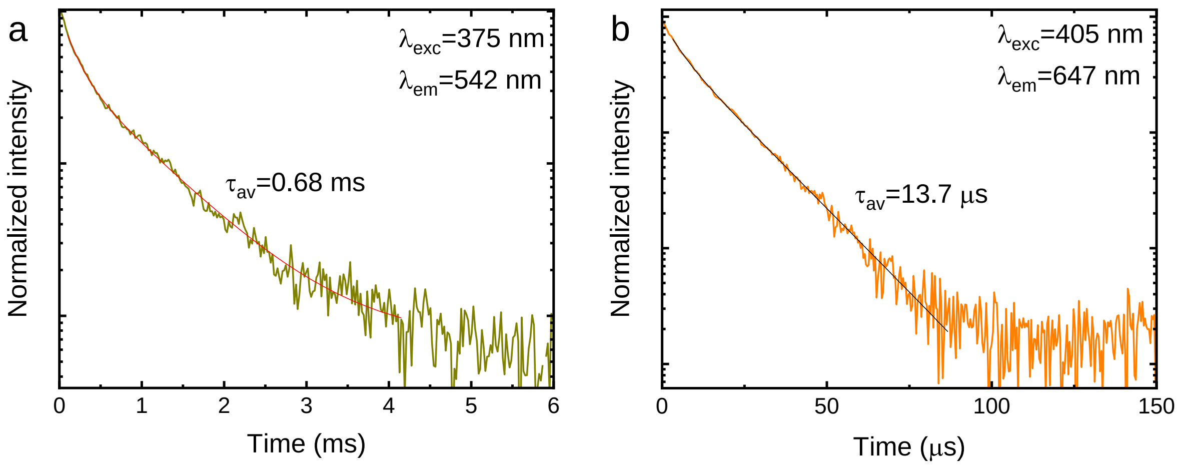

Figure S13. The luminescence decay profiles of **4** (a) and **5** (b) species recorded at room temperature.

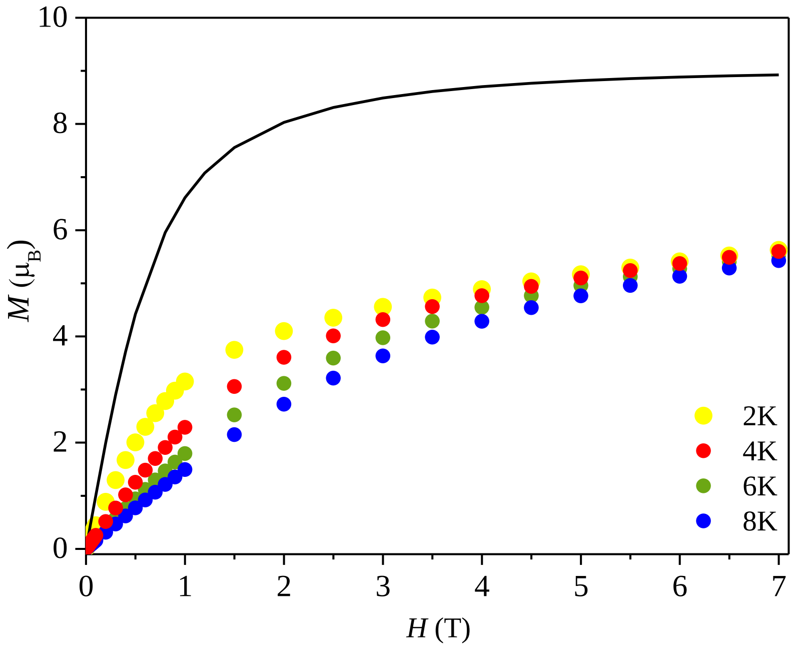

Figure S14. Field dependence of the magnetization for **3** (M per Er^{3+} ion) at 2−8 K. The solid line is the Brillouin function curve for free, non-interacting Er^{3+} ion with $J = {}^{15}/_2$ ground state.

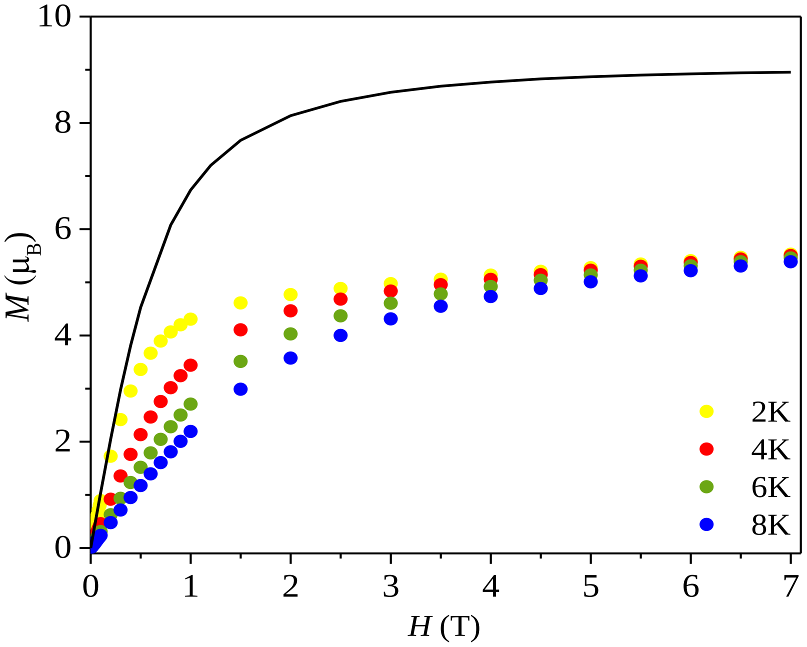

Figure S15. Field dependence of the magnetization for **4** (*M* per Tb^{3+} ion) at 2−8 K. The solid line is the theoretical curve for free, non-interacting Tb^{3+} ion with $J = 6$ ground state.
